# Half-time Tc-99m sestamibi imaging with a direct conversion molecular breast imaging system

**DOI:** 10.1186/2191-219X-4-5

**Published:** 2014-01-15

**Authors:** Carrie B Hruska, Amy Lynn Conners, Katie N Jones, Amanda L Weinmann, Ravi K Lingineni, Rickey E Carter, Deborah J Rhodes, Michael K O’Connor

**Affiliations:** 1 Department of Radiology, Mayo Clinic, 200 First Street SW, Rochester, MN 55905, USA; 2 Department of Health Sciences Research, Mayo Clinic, 200 First Street SW, Rochester, MN 55905, USA; 3 Department of Medicine, Mayo Clinic, 200 First Street SW, Rochester, MN 55905, USA

**Keywords:** Breast cancer, Molecular breast imaging, CZT, Direct conversion, Dose reduction, Wide beam reconstruction

## Abstract

**Background:**

In an effort to reduce necessary acquisition time to perform molecular breast imaging (MBI), we compared diagnostic performance of MBI performed with standard 10-min-per-view acquisitions and half-time 5-min-per-view acquisitions, with and without wide beam reconstruction (WBR) processing.

**Methods:**

Eighty-two bilateral, two-view MBI studies were reviewed. Studies were performed with 300 MBq Tc-99 m sestamibi and a direct conversion molecular breast imaging (DC-MBI) system. Acquisitions were 10 min-per-view; the first half of each was extracted to create 5-min-per-view datasets, and WBR processing was applied.

The 10-min-, 5-min-, and 5-min-per-view WBR studies were independently interpreted in a randomized, blinded fashion by two radiologists. Assessments of 1 to 5 were assigned; 4 and 5 were considered test positive. Background parenchymal uptake, lesion type, distribution of non-mass lesions, lesion intensity, and image quality were described.

**Results:**

Considering detection of all malignant and benign lesions, 5 min-per-view MBI had lower sensitivity (mean of 70% vs. 85% (*p* ≤ 0.04) for two readers) and lower area under curve (AUC) (mean of 92.7 vs. 99.6, *p* ≤ 0.01) but had similar specificity (*p* = 1.0). WBR processing did not alter sensitivity, specificity, or AUC obtained at 5 min-per-view.

Overall agreement in final assessment between 5-min-per-view and 10-min-per-view acquisition types was near perfect (*κ* = 0.82 to 0.89); however, fair to moderate agreement was observed for assessment category 3 (probably benign) (*κ* = 0.24 to 0.48). Of 33 malignant lesions, 6 (18%) were changed from assessment of 4 or 5 with 10-min-per-view MBI to assessment of 3 with 5-min-per-view MBI. Image quality of 5-min-per-view studies was reduced compared to 10-min-per-view studies for both readers (3.24 vs. 3.98, *p* < 0.0001 and 3.60 vs. 3.91, *p* < 0.0001). WBR processing improved image quality for one reader (3.85 vs. 3.24, *p* < 0.0001).

**Conclusions:**

Although similar radiologic interpretations were obtained with 10-min- and 5-min-per-view DC-MBI, resulting in substantial agreement in final assessment, notable exceptions were found: (1) perceived image quality at 5 min-per-view was lower than that for 10-min-per-view studies and (2) in a number of cases, assessment was downgraded from a recommendation of biopsy to that of short interval follow-up.

## Background

Recent advancements in dedicated gamma camera technology to permit imaging at low radiation doses now allow consideration of their use in breast cancer screening. In contrast to breast-specific gamma imaging (BSGI), which uses a single detector comprising a pixelated array of scintillating crystals [1], a new generation of systems now employ a dual-head configuration of solid-state (non-scintillating) detectors [2]. These direct conversion molecular breast imaging (DC-MBI) systems directly convert gamma ray energy to electronic signal, offering improved count sensitivity and energy resolution compared to older generation scintillating systems.

The addition of MBI, performed with 740 MBq (20 mCi) Tc-99 m sestamibi and a cadmium zinc telluride (CZT) DC-MBI system, to screening mammography in women with dense breasts was previously reported. Compared to the performance of incident screening mammography alone, addition of prevalent MBI significantly increased cancer detection rate from 3.2 to 10.7 cancers detected per 1,000 women screened [3]. Importantly, addition of MBI to mammography did not reduce positive predictive value (PPV) compared to that from mammography alone, whereas studies examining addition of whole-breast screening ultrasound to screening mammography in women with dense breasts have shown reduction in PPV [4,5].

Following the implementation of a registered high-sensitivity collimation specifically designed for dual-head DC-MBI systems [6] and a CZT-specific energy acceptance window to capture additional photopeak counts [7], DC-MBI is now routinely performed at our institution with injection of approximately 220 to 300 MBq (6 to 8 mCi) Tc-99 m sestamibi, which corresponds to an effective (whole-body) radiation dose of 1.8 to 2.4 mSv. A similar cancer detection rate of 12.0 per 1,000 has been obtained with addition of this low-dose MBI to screening mammography in women with dense breasts [8]. While MBI’s current effective dose of 1.8 to 2.4 mSv is higher than that from the two-view digital mammography (approximately 0.5 mSv) or two-view digital mammography combined with tomosynthesis (approximately 1.0 to 1.5 mSv) [9], all of these effective doses are at or below annual natural background radiation levels (worldwide average 2.4 mSv, range 1 to 13 mSv) [10]. At these low radiation levels, an assessment of risk has been determined unwarranted, according to recent statements issued by United Nations Scientific Committee on the Effects of Atomic Radiation (UNSCEAR) and American Association of Physicists in Medicine [11,12]. Nevertheless, in an effort to keep doses from medical imaging as low as reasonably achievable and to promote acceptance of MBI in the screening environment, further dose reductions may be desirable.

Recent work showed feasibility of performing MBI with either a further reduced dose of 150 MBq Tc-99 m sestamibi or, alternatively, using a reduced acquisition length of 5minperview while keeping administered dose at 300 MBq [7,13]. With the current 10-min-per-view protocol, a two-view bilateral MBI requires at least 40 min of imaging time. Reduction in the acquisition length of MBI is desirable in order to allow greater throughput, more easily permit the collection of additional views within a single exam time when necessary, and likely make the exam more comfortable for some patients who have difficulty remaining still throughout each 10-min acquisition.

Similar efforts to reduce dose and/or acquisition length for cardiac single photon emission tomography (SPECT) led to the development of a reconstruction technique called wide beam reconstruction (WBR) [14,15]. WBR incorporates the exact collimator geometry, patient-to-detector distance, and statistical characteristics of photon counts to provide both resolution recovery and noise reduction [16]. A modified WBR algorithm has been specifically created for DC-MBI systems.

Our objective was to compare diagnostic performance of three MBI acquisition durations: (a) 10 min-per-view, (b) 5 min-per-view, and (c) 5 min-per-view with WBR processing.

## Methods

### Molecular breast imaging studies

MBI studies were collected under IRB-approved, HIPAA-compliant research protocols and written informed consent was obtained from all participants.

MBI was performed on a DC-MBI system comprising two compact CZT detectors with 1.6 mm × 1.6 mm pixels (LumaGem, Gamma Medica, Salem, NH, USA) and equipped with high sensitivity registered collimators, as previously described [6]. An energy acceptance window of 110 to 154 keV was used [7].

A dispensed dose of 300 MBq (8 mCi) Tc-99 m sestamibi was administered to each patient by intravenous injection. As Tc-99 m sestamibi is known to adhere to plastic walls of syringes, residual dose in the syringe was measured after injection. Approximately 20% residual activity remained in the syringe, on average, giving an average administered dose of 240 MBq (6.5 mCi) [17]. Imaging commenced within 2 to 5 min of injection.

Two images of each breast were acquired with the camera orientation analogous to craniocaudal and mediolateral oblique mammographic views. Acquisitions were a total of 10 min per view, and each was acquired in four 2.5-min frames; the first two frames of each acquisition were summed to create a 5-min-per-view dataset for each patient. A first iteration of the DC-MBI WBR algorithm (UltraSPECT, Haifa, Israel), which incorporated collimator-specific information from both detector views in reconstruction, was applied to 5-min-per-view datasets.

Image count density was measured with a region of interest including all breast tissues, excluding lesions, in craniocaudal views of the left breast of each patient and expressed in counts/cm^2^.

### Reader study case selection

Sample size was selected for determining overall proportion of agreement in disease classification (test positive vs. test negative) between 10-min- and 5-min-per-view MBI. For 5-min-per-view MBI to be considered a viable alternative to 10-min-per-view MBI, agreement in disease classification between the two acquisition types was expected to be 0.90. A non-inferiority margin of 0.10 was proposed as the largest difference from 0.90 that could be observed in order to consider the observed agreement non-inferior to the 0.90 assumption. Assuming *α* level = 0.05, 82 patients with paired 5-min-per-view and 10-min-per-view studies yield 80% statistical power to reject the null hypothesis of inferiority. The study was designed to comprise 40% disease-positive and 60% disease-negative cases (including those with benign lesions) in order to have approximately half of the breasts be positive for a lesion and half negative. This constraint was required to avoid attenuation of the kappa statistic (see statistical methods below). For testing overall diagnostic accuracy, quantified as area under curve (AUC) of the receiver operating characteristic (ROC) curve, this sample size and case mix yielded 90% power to detect at least a 0.2 increase in AUC (from null value of 0.5 to 0.7) at the *α* = 0.05 significance level.

MBI studies were retrospectively selected to reflect the typical range of histopathologies, lesion sizes, lesion uptake intensities, and image count densities observed in clinical practice. Studies with distinguishing features that could be easily recalled, such as very large lesions, breast implants, or artifacts, were not selected. Patients with pathology findings of ductal carcinoma *in situ* or invasive cancer were considered to have positive reference standard. Patients with any other pathology findings or benign imaging findings at >12 months in those not biopsied were considered to have negative reference standard.

### MBI interpretation

Two breast imaging fellowship-trained radiologists with 2 and 3 years of experience interpreting MBI each performed three independent reading sessions, separated by 4 to 6 weeks, of 82 MBI studies at each session. Each session comprised a random order of studies from all three acquisition types (10 min-per-view, 5 min-per-view, or 5 min-per-view with WBR); only one of the three acquisition types appeared for each patient per session. Readers were blinded to acquisition type, other imaging findings, and clinical information.

MBI studies were interpreted according to a validated lexicon for gamma imaging of the breast [18,19]. Background uptake in normal breast parenchyma was described as photopenic, minimal mild, moderate, or marked. Any identified lesions were categorized as mass or non-mass uptake; non-mass uptake distribution was further described as either focal area, segmental, regional, multiple regions, or diffuse. Lesion intensity was qualitatively described as photopenic, mild, moderate, or marked.

Readers assigned a final assessment on a per-breast basis using a 1 to5 scale that parallels BI-RADS assessment categories. Assessments and associated recommendations are as follows: 1 (negative) or 2 (benign, return to routine screening), 3 (probably benign, short-interval follow-up),and 4 (suspicious) or 5 (highly suggestive of malignancy, biopsy) [20]. An overall assessment of diagnostic image quality was assigned, with consideration given to the noise level in the images. Image quality was scored on a 1 to 5 scale as follows: 1 (poor, non-diagnostic); 2 (suboptimal, worse than routine), 3 (acceptable, noisier than routine), 4 (good, same as routine), and 5 (excellent, better than routine).

### Data analysis

Statistical analysis system (SAS) software (SAS Institute, Inc., Cary, NC, USA), version 9.3, was used for all analyses.

Using recommendation of biopsy as the threshold, each breast was classified using a 1 to 5 rating scale. Assessments of 1, 2, and 3 were considered test negative; 4 and 5 were considered test positive. Because lesion detection was the objective of the analysis, sensitivity was calculated two ways: first, considering the sensitivity for detection of all lesions (including malignant and benign) and second, considering sensitivity for detection of cancers only. Sensitivity, specificity, AUC, and average image quality score for each acquisition type were calculated on the per-breast level under the statistical assumption that the individual breast readings were statistically independent. McNemar’s test for correlated proportions was used to compare sensitivities and specificities; Chi-square analysis was used to compare AUCs; and Wilcoxon signed-rank test was used to compare image quality scores between acquisition types. Sensitivities of subgroups within each acquisition type were compared with Fisher’s exact probability test. Two-sided *p* values were reported, and *p* < 0.05 was considered statistically significant.

Proportion of agreement between each individual reader’s interpretation of 10-min-per-view MBI with that of the 5-min-per-view MBI, with and without WBR, was determined for each descriptor. Weighted kappa (*κ*) statistic, which is the proportion of agreement expected beyond chance, was reported on the per-breast level. The scale established by Landis and Koch was followed: *κ* between 0 and 0.2 indicates slight agreement, 0.21 to 0.4 indicates fair agreement, 0.41 to 0.6 indicates moderate agreement, 0.61 to 0.8 indicates substantial agreement, and *κ* above 0.81 indicates near-perfect agreement [21].

Additional lesions described beyond those identified at case selection were not included in agreement analysis. Final assessment categories were combined into three groups for agreement analysis: 1 and 2, 3,and 4 and 5. For determining overall proportion of agreement in disease classification, assessments of 1 to 3 and 4 to 5 were grouped.

## Results

### Characteristics of selected cases

**Figure 1:**
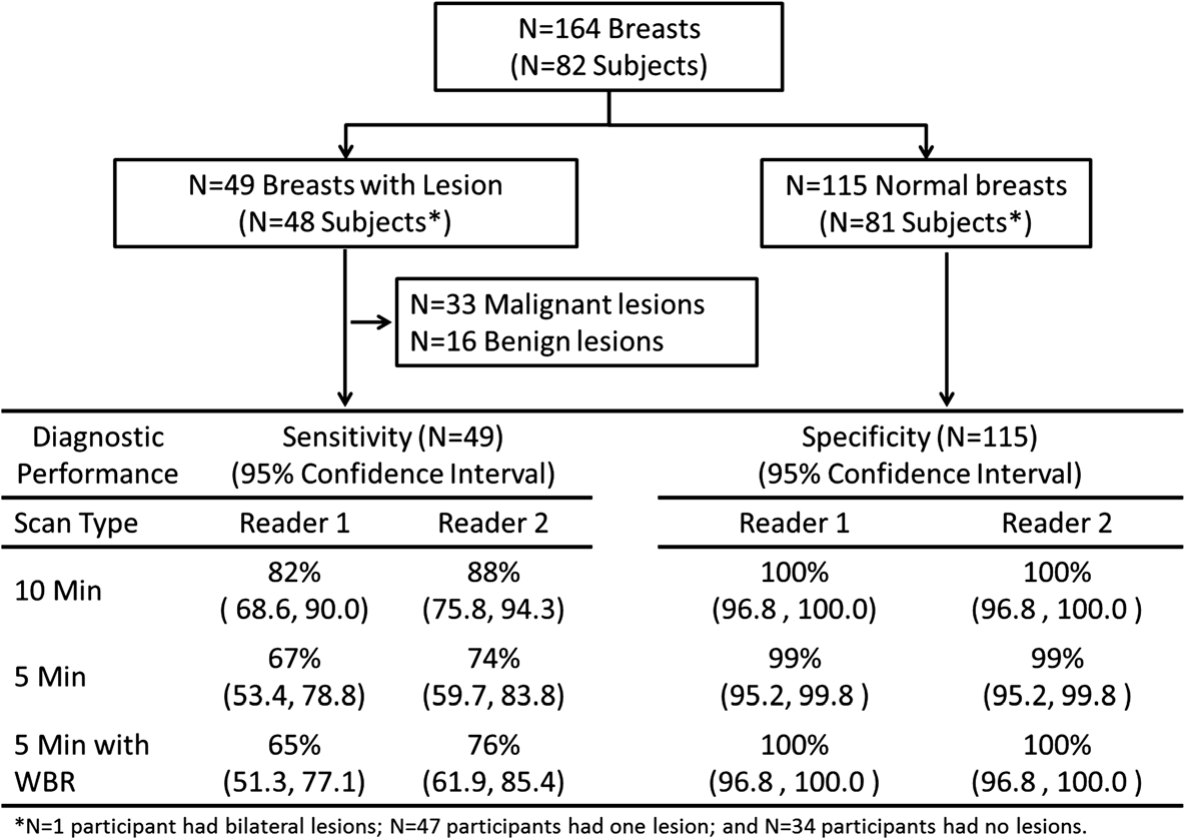
Number of subjects, breasts, and lesions included for sensitivity and specificity analysis.

Average age of patients was 61 years (s.d. 11.2 years; range 41 to 83 years). In the 82 patients, reference standard was positive for breast cancer in 33 and negative in 49. A total of 49 lesions in 48 patients were identified at case selection, including 33 malignant and 16 benign lesions. There were 115 negative breasts for specificity analysis (Figure 1). One patient had a single benign lesion identified in each breast. Characteristics of the lesions are given in Table 1.

**Table 1:** Characteristics of 49 lesions in 48 patients from a total of 82 MBI studies

**Lesion histopathology**	**Number of lesions (% of 49 total lesions)**
Malignant lesions	33 (67)
Invasive ductal carcinoma	23 (47)
Invasive lobular carcinoma	3 (6)
Mixed invasive ductal/lobular	1 (2)
Ductal carcinoma in situ	4 (8)
Mucinous carcinoma	1 (2)
Tubular carcinoma	1 (2)
Benign lesions	16 (33)
Atypia or LCIS	2 (4)
Benign axillary or intramammary lymph node	3 (6)
Fibroadenoma	3 (6)
Papilloma	2 (4)
Benign breast parenchyma/fibrocystic changes	6 (12)
**Lesion size and intensity category**	
**≤** 10 mm and mild intensity	13 (27)
**≤** 10 mm and moderate intensity	4 (8)
**≤** 10 mm and marked intensity	0
**>** 10 mm and mild intensity	5 (10)
**>** 10 mm and moderate intensity	17 (35)
**>** 10 mm and marked intensity	10 (20)

Numbers in parentheses are percentages and percentages are rounded. All patients had a single lesion diagnosed, except for one patient who had a single benign lesion in each breast. Lesion size was the greatest dimension of lesion measured on MBI. Both lesion size and lesion intensity listed were based on a clinically-interpreted MBI report of 10-min-per-view acquisition and used to guide appropriate selection for the reader study.

Median size of all 49 lesions was 1.3 cm (s.d. 0.7 cm; range 0.4 to 3.1 cm) and median size of the 33 malignancies was 1.4 cm (s.d. 0.7 cm; range 0.5 to 3.1 cm). Of 33 malignant lesions, 28 were invasive; of these 29 invasive cancers, 22 were node negative, 5 had positive micrometastasis (N1mi), 1 had a single positive node (N1), and 1 had metastatic breast cancer.

Median image count density of 10-min-per-view MBI studies was 1,574 counts/cm^2^, with a range of 527 to 2,930 counts/cm^2^. In 10-min-per-view studies, 17 of 82 (21%) were considered low count density (<1,150 counts/cm^2^), 38 of 82 (46%) were considered intermediate count density (1,150 to 1,750 counts/cm^2^), and 27 of 82 (33%) were considered high count density (>1,750 counts/cm^2^). Corresponding 5-min-per-view datasets for each patient contained approximately half the count density of the 10-min-per-view acquisitions.

### Diagnostic accuracy

Sensitivity and specificity obtained for each acquisition type is shown in Figure 1. Table 2 expands upon these results for all lesions and malignant lesions only, respectively, by lesion characteristics. Considering detection of all 49 malignant and benign lesions, sensitivity was significantly lower for 5-min-per-view studies compared to 10-min-per-view for both readers; specificity was nearly unchanged. Considering detection of the 33 malignant lesions only, sensitivity was numerically lower and specificity was numerically higher for 5-min-per-view MBI compared to 10-min-per-view MBI; however, differences were not statistically significant. Application of WBR processing did not significantly alter 5-min-per-view MBI sensitivity or specificity.

**Table 2:** Diagnostic performance of each type of MBI acquisition

	**Reader 1**									**Reader 2**								
	**10 min-per-view**		**5 min-per-view**		**5 min-per-view with WBR**		***p *** value*			**10 min-per-view**		**5 min-per-view**		**5 min-per-view with WBR**		***p *** value*		
							**10 min vs. 5 min**	**10 min vs. 5 min WBR**	**5 min vs. 5 min WBR**							**10 min vs. 5 min**	**10 min vs. 5 min WBR**	**5 min vs. 5 min WBR**
	**No. of breasts**	**95% CIs**	**No. of breasts**	**95% CIs**	**No. of breasts**	**95% CIs**				**No. of breasts**	**95% CIs**	**No. of breasts**	**95% CIs**	**No. of breasts**	**95% CIs**			
Detection of both malignant and benign lesions																		
Sensitivity for all lesions***	40/49 (82)	68.6, 90.0	33/49 (67)	53.4, 78.8	32/49 (65)	51.3, 77.1	0.04	0.02	1.00	43/49 (88)	75.8, 94.3	36/49 (74)	59.7, 83.8	37/49 (76)	61.9, 85.4	0.02	0.03	1.00
Lesions ≤10 mm	13/17 (77)	52.7, 90.4	9/17 (53)	31.0, 73.8	7/17 (41)	21.6, 64.0	0.13	0.03	0.50	12/17 (71)	46.9, 86.7	8/17 (47)	26.2, 69.0	9/17 (53)	31.0, 73.8	0.13	0.25	1.00
Lesions >10 mm	27/32 (84)	68.2, 93.1	24/32 (75)	57.9, 86.7	25/32 (78)	61.2, 89.0	0.38	0.63	1.00	31/32 (97)	84.3, 99.4	28/32 (88)	71.9, 95.0	28/32 (88)	71.9, 95.0	0.25	0.25	1.00
*p* value ≤ 10 mm vs. > 10 mm**	0.70		0.20		0.01					0.02		0.01		0.01				
Mild intensity lesions	11/18 (61)	38.6, 79.7	5/18 (28)	12.5, 50.9	4/18 (22)	9.0, 45.2	0.03	0.02	1.00	12/18 (67)	43.7, 83.7	7/18 (39)	20.3, 61.4	7/18 (39)	20.3, 61.4	0.06	0.06	1.00
Moderate/Marked intensity lesions	29/31 (94)	79.3, 98.2	28/31 (90)	75.1, 96.7	28/31 (90)	75.1, 96.7	1.00	1.00	NDP	31/31 (100)	89.0, 100	29/31 (94)	79.3, 98.2	30/31 (97)	83.8, 99.4	NDP	NDP	1.00
*p* value, mild vs. moderate/high intensity**	0.01		<0.001		<0.001					0.001		<0.001		<0.001				
Low image count density	7/8 (88)	52.9, 97.8	7/8 (88)	52.9, 97.8	6/8 (75)	40.9, 92.9	NDP	1.00	1.00	7/8 (88)	52.9, 97.8	6/8 (75)	40.9, 92.9	7/8 (88)	52.9, 97.8	1.00	NDP	1.00
Intermediate/high image count density	33/41 (81)	66.0, 89.8	26/41 (63)	48.1, 76.4	26/41 (63)	48.1, 76.4	0.04	0.04	1.00	36/41 (88)	74.5, 94.7	30/41 (73)	58.1, 84.3	30/41 (73)	58.1, 84.3	0.03	0.03	1.00
*p* value, low vs. intermediate/high image count density**	1.00		0.25		0.70					1.00		1.00		0.66				
Specificity	115/115 (100)	96.8, 100.0	114/115 (99)	95.2, 99.8	115/115 (100)	96.8, 100.0	1.00	NDP	1.00	115/115 (100)	96.8, 100.0	114/115 (99)	95.2, 99.8	115/115 (100)	96.8, 100.0	1.00	NDP	1.00
	**AUC**	**95% CIs**	**AUC**	**95% CIs**	**AUC**	**95% CIs**				**AUC**	**95% CIs**	**AUC**	**95% CIs**	**AUC**	**95% CIs**			
ROC analysis	99.4	98.8, 99.9	91.9	86.5, 97.4	93.8	89.1, 98.4	0.01	0.02	0.39	99.8	99.6, 100.0	93.4	88.5, 98.2	96.0	92.2, 99.8	0.01	0.04	0.23
Detection of malignant lesions only																		
Sensitivity for malignant lesions	29/33 (88)	72.7, 95.2	26/33 (79)	62.2, 89.3	27/33 (82)	65.6, 91.4	0.25	0.50	1.00	31/33 (94)	80.4, 98.3	29/33 (88)	72.7, 95.2	28/33 (85)	69.1, 93.3	0.50	0.25	1.00
Cancers ≤10 mm	6/8 (75)	40.9, 92.9	5/8 (63)	30.6, 86.3	5/8 (63)	30.6, 86.3	1.00	1.00	NDP	6/8 (75)	40.9, 92.9	5/8 (63)	30.6, 86.3	5/8 (63)	30.6, 86.3	1.00	1.00	NDP
Cancers >10 mm	23/25 (92)	75.0, 97.8	21/25 (84)	65.3, 93.6	22/25 (88)	70.0, 95.8	0.50	1.00	1.00	25/25 (100)	86.7, 100	24/25 (96)	80.5, 99.3	23/25 (92)	75.0, 97.8	1.00	1.00	1.00
*p* value ≤ 10 mm vs. > 10 mm**	0.24		0.32		0.14					0.053		0.036		0.080				
Mild intensity cancers	5/9 (56)	26.7, 81.1	3/9 (33)	12.1, 64.6	4/9 (44)	18.9, 73.3	0.50	1.00	1.00	7/9 (78)	45.3, 93.7	6/9 (67)	35.4, 87.9	4/9 (44)	18.9, 73.3	1.00	0.25	0.50
Moderate/marked intensity cancers	24/24 (100)	86.2, 100	23/24 (96)	79.8, 99.3	23/24 (96)	79.8, 99.3	1.00	1.00	NDP	24/24 (100)	86.2, 100	23/24 (96)	79.8, 99.3	24/24 (100)	86.2, 100	1.00	1.00	1.00
*p* value, mild vs. moderate/high intensity**	0.003		< 0.001		0.003					0.068		0.052		< 0.001				
Low image count density	6/7 (86)	48.7, 97.4	6/7 (86)	48.7, 97.4	6/7 (86)	48.7, 97.4	NDP	NDP	NDP	6/7 (86)	48.7, 97.4	6/7 (86)	48.7, 97.4	6/7 (86)	48.7, 97.4	NDP	NDP	NDP
Intermediate/high image count density	23/26 (89)	71.0, 96.0	20/26 (77)	58.0, 89.0	21/26 (81)	62.1, 91.5	0.25	0.50	1.00	25/26 (96)	81.1, 99.3	23/26 (89)	71.0, 96.0	22/26 (85)	66.5, 93.8	0.50	0.25	1.0
*p* value, low vs. intermediate/high image count density**	1.00		1.00		1.00					0.38		1.00		1.00				
Specificity	120/131 (92)	85.6, 95.2	123/131 (94)	88.4, 96.9	126/131 (96)	91.4, 98.4	0.45	0.07	0.25	119/131 (91)	84.7, 94.7	123/131 (94)	88.4, 96.9	122/131 (93)	87.5, 96.3	0.22	0.25	1.00
	**AUC**	**95% CIs**	**AUC**	**95% CIs**	**AUC**	**95% CIs**				**AUC**	**95% CIs**	**AUC**	**95% CIs**	**AUC**	**95% CIs**			
ROC analysis	96.0	93.6, 98.3	95.0	90.9, 99.1	96.9	94.3, 99.0	0.56	0.45	0.21	96.1	93.9, 98.3	94.9	90.8, 98.9	94.7	90.6, 98.7	0.47	0.38	0.93

Diagnostic performance of each type of MBI acquisition interpreted independently by two readers, first considering detection of both malignant and benign lesions and second considering detection of only malignant lesions. Numbers in parentheses are percentages, and percentages are rounded.MBI, molecular breast imaging; WBR, wide beam reconstruction; NDP, unable analyze due to lack discordant pairs. A per-breast final assessment of 1, 2, or 3 was considered test negative, and final assessment of 4 or 5 was considered test positive. Lesion size and intensity classification were determined at case selection based on the clinically interpreted MBI report of the 10-min-per-view acquisitions. Image count density was measured in the background (non-lesion) region of benign breast tissue on the 10-min-per-view MBI acquisition. Count density was defined as low (<1,150 counts/cm^2^), intermediate (1,150 to 1,750 counts/cm^2^), or high (>1,750 counts/cm^2^). *p values for sensitivity and specificity were calculated using McNemar’s test for correlated proportions, *p* values for ROC results were calculated using Chi-square analysis, and *p* values for image quality scores were calculated using Wilcoxon signed-rank test. **p values were calculated using Fisher’s exact probability test. ***Results for sensitivity for all lesions are given for 49 breasts with lesions (33 malignant and 16 benign) in 48 patients. One patient had a single benign lesion in each breast.

Sensitivity for lesion detection was impacted by lesion size and intensity (Table 2). Sensitivity for lesions ≤10 mm was consistently lower than that for cancers larger than 10 mm for all acquisition types and significantly lower for reader 2. Sensitivity for mild-intensity lesions was significantly lower than that for moderate or marked intensity lesions for all acquisition types for both readers. No significant difference in sensitivity was observed between studies with starting low image count density at 10 min-per-view compared to studies with starting intermediate or high image count density for any acquisition type. However, the effect of image count density on lesion detection is reflected in the decreased sensitivity for lesion detection with proportional halving of the count density as image acquisition duration was halved from 10 to 5 min-per-view.

When considering detection of all lesions, ROC analysis demonstrated a high AUC of above 91 for all acquisition types (Table 2); however, AUC was significantly lower at 5 min-per-view compared to 10 min-per-view for both readers. The application of WBR processing did not significantly alter the AUC obtained with 5-min-per-view MBI.

**Figure 2:**
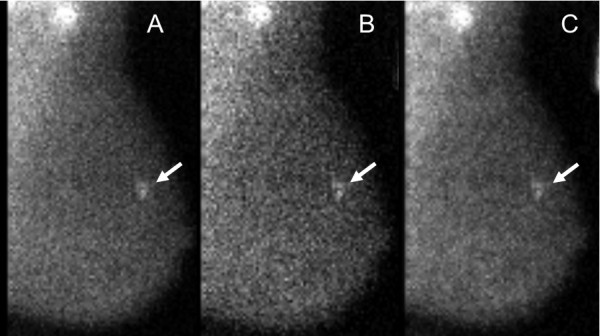
**MBI study performed with injection of 290 MBq Tc-99 m sestamibi and a dual-head DC-MBI system.** In this 63-year-old woman, a 0.8-cm ductal carcinoma *in situ* appears as a moderate intensity mass (arrow) and was detected at all three acquisition types: **(A)** 10 min-per-view, **(B)**5 min-per-view, and **(C)** 5 min-per-view with WBR. Image count density of 1,020 counts/cm^2^ in the 10-min-per-view acquisition was considered low. Both readers assigned a test positive assessment of 4 (suspicious) for all three acquisition types.

**Figure 3:**
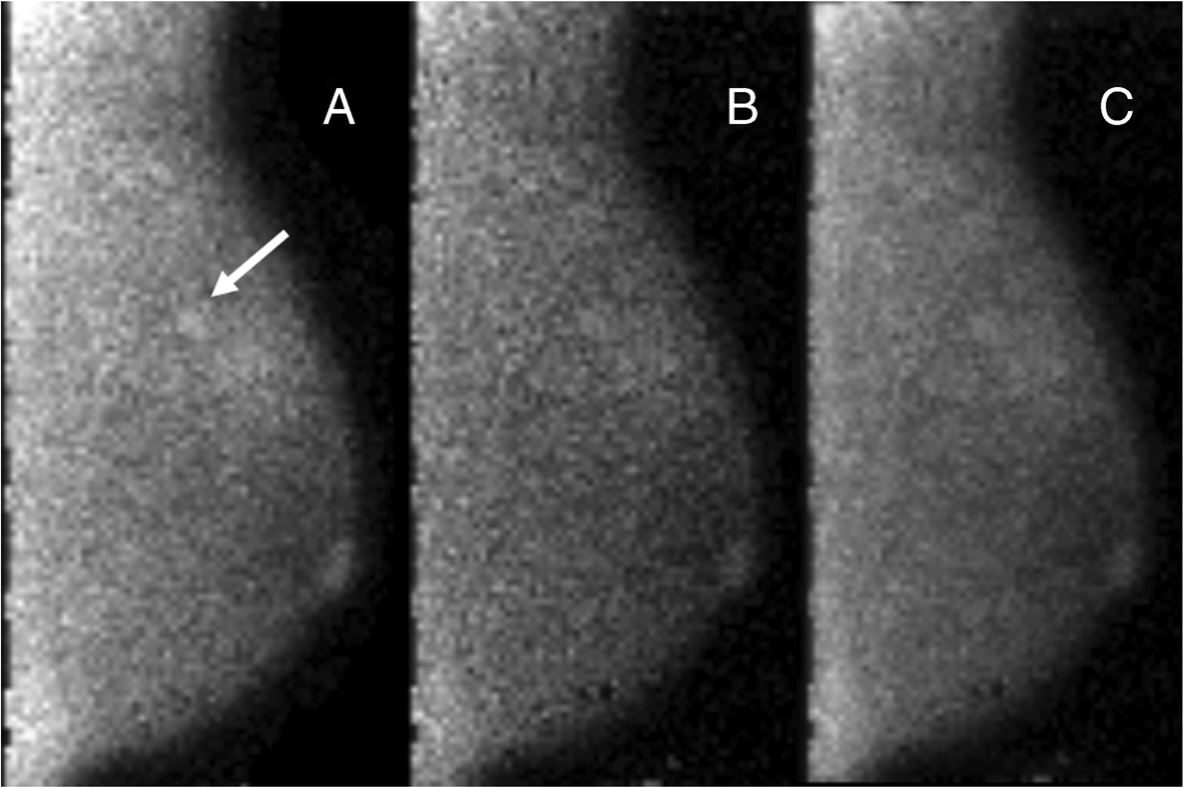
**MBI study performed with injection of 320 MBq Tc-99 m sestamibi and a dual-head DC-MBI system.** In this 45-year-old woman, a 0.5-cm invasive ductal carcinoma appears as a mild intensity, focal area of non-mass uptake (arrow). Image count density of 1,800 counts/cm^2^ in the 10-min-per-view acquisition was considered high. The cancer was detected at **(A)** 10-min-per-view, but not at **(B)** 5 min-per-view or **(C)** 5 min-per-view with WBR. At 10 min-per-view, one reader assigned a test positive assessment of 4 (suspicious), and the other reader gave a test negative assessment of 3 (probably benign). For both 5 min-per-view and 5-min-per-view WBR acquisitions, both readers assigned test negative assessments of 2 (benign). These images correspond with patient no. 4 in Table 3.

Example images of the three acquisition types in patients are shown in Figures 2 and 3.

### Changes in final assessment score

In 15 patients, a lesion that was detected (assessment 4 or 5) on 10-min-per-view MBI by either reader was given a test negative assessment of 1 to 3 on the 5 min-per-view or 5-min-per-view WBR study by either one or both readers (Table 3). The 15 lesions included 6 malignancies and 9 benign lesions. All were mild intensity except for one of the invasive ductal carcinomas which was moderate intensity; size ranged from 0.5 to 2.5 cm.

**Table 3:** Lesions that were test positive on 10-min-per-view MBI but test negative on 5-min-per-view MBI

	**Lesion histopathology**	**Size (cm)**	**Grade**	**Node status**	**Lesion intensity**	**Image count density**	**Reader 1**		**Reader 2**	
							**5-min-per-view assessment**^**a**^	**5-min-per-view WBR assessment**^**a**^	**5-min-per-view assessment**^**a**^	**5-min-per-view WBR assessment**^**a**^
Patient number										
1	Invasive ductal carcinoma	0.9	III	N0	Mild	High	3	3	1	3
2	Invasive ductal carcinoma	1.8	II	N0	Moderate	Intermediate	3	3	3	(4)
3	Invasive ductal carcinoma	2.5	I	N1mi	Mild	Intermediate	3	(4)	(4)	(4)
4	Invasive ductal carcinoma	0.5	III	N1	Mild	High	2	2	2	2
5	Invasive ductal carcinoma	2.0	II	N0	Mild	Intermediate	3	3	(4)	3
6	Mucinous carcinoma	1.1	I	N0	Mild	Intermediate	3	3	(4)	3
7	Atypical ductal hyperplasia	0.9	-	-	Mild	Intermediate	(4)	3	3	3
8	Benign fibrocystic changes	0.4	-	-	Mild	Intermediate	1	1	1	1
9	Benign fibrocystic changes	1.8	-	-	Moderate	High	3	3	(4)	3
10	Fibroadenoma	0.8	-	-	Mild	High	2	1	1	2
11	Fibroadenoma	1.0	-	-	Mild	Intermediate	1	3	3	3
12	Benign breast parenchyma	3.0	-	-	Mild	High	1	1	1	3
13	Benign lymph node	0.8	-	-	Mild	Low	(4)	3	3	(4)
14	Benign breast parenchyma	1.1	-	-	Mild	High	1	3	3	(4)
15	Benign lymph node	1.5	-	-	Moderate	Intermediate	3	3	3	(4)
No. of patients with assessment 3							7	10	6	7
No. of patients with assessment 1 or 2							6	4	5	3

Assessments of 4 or 5 were considered test positive; assessments of ≤3 were considered test negative. ^a^Final assessment is given for lesions detected by either reader on 10-min-per-view acquisitions but not detected by at least one reader on 5 min-per-view or 5-min-per-view WBR acquisitions. Assessments in parentheses indicate a detected lesion. Lesion size and intensity classification was determined at case selection based on the clinically-interpreted MBI report of the 10-min-per-view acquisitions. Image count density was measured in the background (non-lesion) region of benign breast tissue on the 10-min-per-view MBI acquisition. Count density was defined as low (<1,150 counts/cm^2^), intermediate (1,150 to1,750 counts/cm^2^), or high (>1,750 counts/cm^2^). Note: in one additional patient, a benign lesion was considered undetected at 10 min-per-view (assessment = 3) but was detected at 5 min-per-view and 5 min-per-view with WBR (assessment of 4) by reader 1.

Of all 15 lesions with downgraded assessments at 5 min-per-view, 12 of 15 (80%) were categorized as mild intensity lesions and the other 3 of 15 (20%) were moderate intensity. Approximately half (7 of 15 (47%)) of these lesions were ≤10 mm. Only 1 of 15 (7%) was in a patient with a low image count density. Eleven of the 15 (73%) test-negative lesions on 5 min-per-view or 5-min-per-view WBR acquisitions received a final assessment of 3 (probably benign) by either one or both readers, indicating that the lesion was observed but not given a test positive assessment of 4 or 5 that would lead to recommendation of biopsy.

For reader 1, the application of WBR to the 5-min-per-view acquisitions resulted in an upgrade from a test negative to test positive assessment in only one lesion, a malignant lesion in patient no. 3, and a downgraded assessment from test positive to test negative in 2 benign lesions (patient nos. 7 and 13). For reader 2, application of WBR resulted in an upgrade from test negative to test positive in four lesions: one malignancy in patient no. 2 and three benign lesions in patient nos. 13 to 15; however, two malignant lesions (patient nos.5 and 6) and one benign lesion (patient no. 9) were downgraded from test positive to test negative.

### Image quality

For both readers, average image quality scores were significantly lower for 5-min-per-view acquisitions compared to 10-min-per-view acquisitions (3.24 vs. 3.98, *p* < 0.0001 and 3.60 vs. 3.91, *p* < 0.0001) as shown in Table 4. For reader 1, application of WBR led to improvements in 5-min-per-view image quality (3.85 vs. 3.24, *p* < 0.0001), resulting in a score similar to 10-min-per-view studies. However, reader 2 results did not show improvement in image quality with WBR.

**Table 4:** Image quality scores for each MBI acquisition type interpreted independently by two readers

	**10 min-per-view**		**5 min-per-view**		**5 min-per-view with WBR**		***p *** value*		
							**10 min vs. 5 min**	**10 min vs. 5 min WBR**	**5 min vs. 5 min WBR**
	**Average**	**S.D.**	**Average**	**S.D.**	**Average**	**S.D.**			
**Reader 1**	3.98	0.42	3.24	0.66	3.85	0.45	<0.0001	0.086	<0.0001
**Reader 2**	3.91	0.28	3.60	0.61	3.49	0.63	<0.0001	<0.0001	0.17

No studies of any acquisition type received an image quality score of 1. Of the 82 studies performed at 10 min-per-view, only one received an image quality score of 2 from one reader; image count density in that study was 739 counts/cm^2^. In the corresponding 82 studies at 5 min-per-view, a total of 12 patients were assigned with image quality scores of 2 by the combination of results from both readers; average image count density in those studies was 612 counts/cm^2^, ranging from 264 to 1,009 counts/cm^2^.

### Interpretation agreement

Agreement of 10-min-per-view interpretations with 5-min-per-view interpretations is given in Table 5. Overall proportion of agreement in disease classification between 10 min- and 5-min-per-view MBI, determined by grouping test negative (1 to 3) and test positive (4 to 5) assessments, was 154 of 164 (94%) for reader 1 and 156 of 164 (95%) for reader 2.

**Table 5:** Agreement between each reader’s interpretation of 5-min-per-view and 10-min-per-view MBI

	**Reader 1**					**Reader 2**				
		**10 min-per-view vs. 5 min-per-view**		**10-min-per-view vs. 5-min-per-view WBR**			**10-min-per-view vs. 5-min-per-view**		**10-min-per-view vs. 5-min-per-view WBR**	
	**No. of breasts**	**Overall agreement (95% CI)**	***κ*(95% CI)**	**Overall agreement (95% CI)**	***κ*(95% CI)**	**No. of breasts**	**Overall agreement (95% CI)**	***κ*(95% CI)**	**Overall agreement (95% CI)**	***κ*(95% CI)**
Background parenchymal uptake		151/164 (92) (87, 95)	0.84 (0.76, 0.92)	157/164 (96) (91, 98)	0.89 (0.81, 0.98)		150/164 (91) (86, 95)	0.79 (0.69, 0.89)	150/164 (91) (86, 95)	0.82 (0.73, 0.91)
Photopenic	24		0.87 (0.75, 0.98)		0.87 (0.75, 0.98)	22		0.82 (0.68, 0.96)		0.77 (0.62, 0.92)
Mild	124		0.84 (0.75, 0.94)		0.92 (0.84, 0.99)	128		0.81 (0.70, 0.92)		0.79 (0.67, 0.90)
Moderate	8		0.47 (0.16, 0.78)		0.79 (0.59, 0.99)	8		0.58 (0.31, 0.84)		0.65 (0.39, 0.91)
Marked	8		0.74 (0.49, 0.98)		1.00 (1.00, 1.00)	6		0.49 (0.07, 0.92)		0.85 (0.65, 1.00)
Lesion type		138/164 (84) (78, 89)	0.67 (0.56, 0.78)	142/164 (87) (81, 91)	0.74 (0.63, 0.84)		147/164 (90) (84, 93)	0.76 (0.65, 0.87)	150/164 (91) (86, 95)	0.78 (0.68, 0.88)
No lesion	107		0.75 (0.64, 0.85)		0.83 (0.74, 0.92)	113		0.80 (0.70, 0.90)		0.81 (0.71, 0.91)
Mass	23		0.61 (0.42, 0.80)		0.69 (0.53, 0.86)	11		0.85 (0.68, 1.00)		0.81 (0.62, 0.99)
Non-mass	34		0.56 (0.40, 0.72)		0.61 (0.45, 0.76)	40		0.72 (0.59, 0.84)		0.74 (0.62, 0.86)
Distribution of non-mass lesions		137/164 (84) (77, 88)	0.46 (0.32, 0.61)	139/164 (85) (78, 89)	0.52 (0.37, 0.66)		147/164 (90) (84, 93)	0.68 (0.54, 0.82)	149/164 (91) (85, 94)	0.70 (0.57, 0.84)
No non-mass lesion	130		0.56 (0.40, 0.72)		0.61 (0.45, 0.76)	124		0.72 (0.59, 0.84)		0.74 (0.64, 0.86)
Focal area	30		0.48 (0.31, 0.66)		0.52 (0.35, 0.69)	38		0.75 (0.62, 0.87)		0.74 (0.61, 0.86)
Segmental	2		−0.01 (−0.02,0.00)		0.66 (0.05, 1.00)	1		0.00 (0.00, 0.00)		0.00 (0.00, 0.00)
Regional	2		−0.02(−0.03,0.00)		−0.01 (−0.03,0.00)	1		0.00 (0.00, 0.00)		0.00 (0.00, 0.00)
Multiple regions	0		N/A		N/A	0		N/A		N/A
Diffuse	0		N/A		N/A	0		N/A		N/A
Lesion intensity		139/164 (85) (78, 89)	0.81 (0.73, 0.89)	145/164 (88) (83, 93)	0.85 (0.77, 0.92)		143/164 (87) (81, 91)	0.80 (0.71, 0.88)	146/164 (89) (83, 93)	0.85 (0.78, 0.92)
No lesion	107		0.76 (0.65, 0.87)		0.83 (0.74, 0.92)	114		0.78 (0.68, 0.89)		0.87 (0.79, 0.95)
Photopenic	2		−0.01 (−0.02,0.00)		1.00 (1.00, 1.00)	0		N/A		N/A
Mild	20		0.46 (0.24, 0.67)		0.58 (0.39, 0.78)	31		0.63 (0.47, 0.78)		0.68 (0.53, 0.83)
Moderate	22		0.73 (0.57, 0.89)		0.77 (0.62, 0.92)	10		0.68 (0.44, 0.92)		0.53 (0.28, 0.77)
Marked	13		0.86 (0.70, 1.00)		0.83 (0.67, 0.99)	9		0.87 (0.69, 1.00)		0.94 (0.82, 1.00)
Final assessment of three groups		144/164 (88) (82, 92)	0.81 (0.73, 0.89)	144/164 (88) (82, 92)	0.82 (0.75, 0.90)		146/164 (89) (83, 93)	0.84 (0.76, 0.91)	151/164 (92) (87, 95)	0.89 (0.82, 0.95)
Assessment 1 or 2	107		0.80 (0.70, 0.90)		0.82 (0.73, 0.91)	112	0.92	0.81 (0.72, 0.91)		0.87 (0.79, 0.95)
Assessment 3	17		0.38 (0.14, 0.62)		0.48 (0.27, 0.69)	9	0.91	0.24 (−0.02,0.50)		0.44 (0.17, 0.72)
Assessment 4 or 5	40		0.83 (0.72, 0.93)		0.82 (0.72, 0.93)	43	0.95	0.87 (0.78, 0.96)		0.90 (0.82, 0.98)
Disease classification		154/164 (94) (89, 97)	0.83 (0.72, 0.93)	154/164 (94) (89, 97)	0.82 (0.72, 0.93)		156/164 (95) (90, 98)	0.87 (0.78, 0.96)	158/164 (96) (92, 98)	0.90 (0.82, 0.98)
Test negative: assessment 1 to 3	124		0.83 (0.72, 0.93)		0.82 (0.72, 0.93)	121	0.95	0.87 (0.78, 0.96)		0.90 (0.82, 0.98)
Test positive: assessment 4 to 5	40		0.83 (0.72, 0.93)		0.82 (0.72, 0.93)	43	0.95	0.87 (0.78, 0.96)		0.90 (0.82, 0.98)
Image quality		55/164 (34) (27, 41)	0.04 (−0.03, 0.11)	126/164 (77) (70, 83)	0.12 (−0.01, 0.26)		116/164 (71) (63, 77)	0.21 (0.09, 0.32)	92/164 (56) (48, 63)	0.04 (−0.04, 0.13)
1 - Poor, non-diagnostic	0		N/A		N/A	0		N/A		N/A
2 - Suboptimal, worse than routine	2		0.16 (−0.04,0.36)		−0.02 (−0.03,0.01)	0		0.00 (0.00, 0.00)		0.00 (0.00, 0.00)
3 - Acceptable, noisier than routine	11		−0.06 (−0.14,0.01)		0.13 (−0.08,0.33)	14		0.23 (0.08, 0.38)		0.03 (−0.08,0.14)
4 - Good, same as routine	141		−0.03 (−0.12,0.05)		0.17 (−0.02,0.36)	150		0.24 (0.11, 0.37)		0.05 (−0.05,0.15)
5 - Excellent, better than routine	10		0.00 (0.00, 0.00)		−0.02 (−0.05,0.00)	0		N/A		N/A

Data are for 82 patients with 164 breasts. Unless otherwise noted, numbers in parentheses are percentages, and percentages are rounded. Number of breasts is those with given feature according to the readers’ interpretations of 10-min-per-view MBI.N/A, kappa statistic was not applicable as no breasts were assigned the given descriptor for any MBI acquisition type.

Overall kappa statistic calculations indicated substantial to near-perfect agreement of 5-min-per-view studies (with and without WBR) and 10-min-per-view studies in describing background parenchymal uptake (*κ* = 0.79 to 0.89), lesion type (*κ* = 0.67 to 0.78), lesion intensity (*κ* = 0.80 to 0.85), and final assessment (*κ* = 0.82 to 0.89). Moderate to substantial overall agreement was observed in describing the distribution of non-mass lesions (*κ* = 0.46 to 0.70). For the final assessment descriptor, substantial to near-perfect agreement was observed for the grouped assessment categories of 1/2 and 4/5, but only fair to moderate agreement was observed for the middle assessment category of 3.

Description of image quality showed the lowest agreement; slight to fair overall agreement (*κ* = 0.04 to 0.21) was observed for both readers.

## Discussion

MBI performed at 5 min-per-view demonstrated decreased sensitivity for lesion detection, similar specificity, and decreased AUC compared to standard 10-min-per-view MBI. According to criteria for non-inferiority established *a priori*, where ‘non-inferior’ was defined as no more than 10% reduction in the expected 90% agreement in disease classification (test negative vs. test positive), 5-min-per-view MBI performance is consistent with the definition of being non-inferior to 10-min-per-view MBI as 94% to 95% overall agreement between the two acquisition types was observed. However, due to the significantly lowered sensitivity, coupled with lower perceived image quality scores and a substantial number of downgraded assessments at 5 min-per-view, the standard 10-min-per-view acquisitions for DC-MBI are preferred.

In general, a high level of agreement was observed between interpretations of 10-min- and 5-min-per-view acquisitions. However, the fair to moderate agreement observed for assessment category 3 is of clinical importance. Similar to other retrospective reader study designs, only assessments with recommendation of biopsy (4 or 5) were considered test positive. Between the two readers, downgrades from assessment 4 to 5 on 10-min-per-view MBI to assessment 3 on 5-min-per-view MBI were observed in 6 of 33 (18%) patients with breast cancer and 9 of 16 (56%) patients with benign lesions. These downgrades would result in changing recommendation from biopsy to short interval MBI follow-up at 6 months.

The six malignant cases that were test negative on 5-min-per-view MBI all presented as focal areas of non-mass uptake. Five were mild intensity and one was moderate intensity. Mild intensity non-mass lesions represent the most subtle of MBI-detectable findings and are often not visible on all views. On subsequent review of these cases, four were seen on a single view on a single projection only (i.e., were visible on only one of four images provided for that breast). Because focal area non-mass lesions are on the edge of MBI detectability, it is not surprising that they were most affected by a decrease in count density by halving acquisition duration in this study.

Lesion intensity was identified as an important factor in determining lesion detection for a given acquisition type, as mild intensity lesions were less likely to be detected. Lesion size also impacted lesion detection, with significantly lower sensitivity observed for lesions ≤10 mm for one reader.

Reduction in acquisition length by half results in half the count density and a corresponding increase in noise fraction by a factor of √2. Of lesions that were test positive on 10 min-per-view, but test negative at 5 min-per-view (Table 3), only one was in a low count density study.

Moderate or marked background parenchymal uptake on MBI can obscure breast lesions. A limitation of this study was that most patients had either photopenic or mild background uptake; only four patients (eight breasts) were assigned moderate or marked background uptake. All lesions downgraded at 5 min-per-view (Table 3) were in patients with photopenic or mild background uptake. Hence, the effect of moderate or marked background parenchymal uptake on lesion detection at 5 min-view could not be assessed in this study.

An additional limitation was that the detection task was a combined detection and characterization process. Lesions, when identified, were done so using the BI-RADS-like assessment scale at the breast level. A subtlety of this analysis is that breasts characterized as 3 were treated as screen failures (MBI negative as a test result with a binary decision).

Application of the current DC-MBI WBR algorithm to 5-min-per-view studies did not significantly impact diagnostic accuracy. WBR processing did result in better perceived image quality for one reader. The usefulness of WBR may therefore depend on reader preference for image appearance. While WBR reduces appearance of image noise, it may also create the perception of loss in overall image contrast. In practice, we anticipate offering WBR images as an adjunct dataset to unprocessed studies.

## Conclusions

Findings indicate that the standard 10-min-per-view acquisition duration for DC-MBI performed with 300 MBq Tc-99 m sestamibi is preferred to 5-min-per-view acquisitions, as sensitivity for lesion detection is reduced with shorter imaging time. Although reduction to 5 min-per-view resulted in similar radiologic interpretations, clinically important exceptions of lower perceived image quality and downgraded assessments from a recommendation of biopsy to that of short interval follow-up were observed.

## Abbreviations

AUC: area under the curve; CZT: cadmium zinc telluride; DC-MBI: direct conversion molecular breast imaging; HIPAA: Health Insurance Portability and Accountability Act; IRB: institutional review board; MBI: molecular breast imaging; PPV: positive predictive value; SPECT: single photon emission tomography; ROC: receiver operator characteristic; WBR: wide beam reconstruction.

## Competing interests

Three authors of this manuscript, Drs. Hruska, Weinmann, and O’Connor receive royalties from licensed technologies per an agreement between Mayo Clinic and Gamma Medica, a company that manufactures the DC-MBI system used in this research. The other authors declare that they have no competing interests.

## Authors’ contributions

CH, DR, and MO were responsible for study concept and design. AC and KJ performed all radiological interpretations. AW assisted with data collection and organization. RL and RC assisted in study design and performed and validated all statistical analyses. All authors read and approved the final version of the manuscript.
